# High-Accuracy Serodiagnosis of African Swine Fever Using P72 and P30-Based Lateral Flow Assays: A Validation Study with Field Samples in Thailand

**DOI:** 10.3390/vetsci13010004

**Published:** 2025-12-19

**Authors:** Nitipon Srionrod, Supphathat Wutthiwitthayaphong, Teera Nipakornpun, Sakchai Ruenphet

**Affiliations:** 1 Clinic for Swine, Mahanakorn University of Technology, 140 Cheum-Sampan Rd., Nong Chock, Bangkok 10530, Thailand; nitipon@mut.ac.th; 2 Animal Biotechnology, Mahanakorn University of Technology, 140 Cheum-Sampan Rd., Nong Chock, Bangkok 10530, Thailand; wsupphathat@mut.ac.th; 3 Pacific Biotech, 42 Moo 4, Petchaboon-Chalianglub Rd., Napa, Muang, Petchaboon 67000, Thailand; teera@brianet.com; 4 Immunology and Virology Department, Mahanakorn University of Technology, Bangkok 10530, Thailand

**Keywords:** African swine fever virus, enzyme-linked immunosorbent assay, lateral flow assay, quantitative polymerase chain reaction

## Abstract

African Swine Fever (ASF) control is severely hampered by the reliance on slow, laboratory-bound diagnostics. While rapid, field-deployable lateral flow assays (LFAs) are urgently needed, the comparative performance of key single-antigen targets remains poorly characterized. This study aimed to develop and systematically evaluate the diagnostic performance of three in-house single-antigen LFAs targeting ASF virus P30, P54, and P72, using swine field samples from Thailand, including a panel of 143 quantitative polymerase chain reaction-negative swine serum samples. The performance of each LFA was compared against a commercial multi-antigen (P32/P62/P72) indirect ELISA, which served as the reference standard, classifying 64 samples as positive and 79 as negative. The P72-based LFA demonstrated perfect diagnostic performance (100% sensitivity, 100% specificity) and perfect agreement (κ = 1.0) with the enzyme-linked immunosorbent assay (ELISA). Similarly, the P30 LFA demonstrated high performance (100% sensitivity, 98.7% specificity) with ‘Almost Perfect’ agreement (κ = 0.9859). In contrast, the P54 LFA was unsuitable, achieving 100% sensitivity but unacceptably low specificity (88.6%) due to a high rate of false positives. Overall, the single-antigen P72 and P30 LFAs demonstrated excellent concordance with the multi-antigen ELISA, supporting their reliable for detecting antibodies against ASFV. Although these assays do not replace molecular methods for acute infection detection, they represent valuable complementary tools for serosurveillance.

## Simple Summary

African Swine Fever is a highly destructive disease devastating pig populations and causing severe economic damage globally. A major challenge in controlling its spread is the reliance on slow, expensive laboratory tests, which delay critical containment efforts. This study aimed to develop and validate simple, rapid “strip tests” (lateral flow assays) that could quickly detect antibodies to the virus at the farm. We created three test prototypes, each targeting a different viral protein (P72, P30, or P54), and tested them against 143 pig serum samples from Thailand. Our results showed that the rapid test targeting P72 was perfectly accurate, matching the complex laboratory test in every case. The P30 test was also found to be highly reliable, while the P54 test proved unsuitable due to a high number of false positive results. We conclude that the P72 and P30 rapid tests are excellent, low-cost tools for surveillance. Their value lies in allowing veterinarians and farmers to obtain accurate results “pen-side” in under 20 min to identify animals with past exposure, enabling immediate action to control outbreaks and protect the pork industry.

## 1. Introduction

African Swine Fever (ASF) is one of the most formidable and economically devastating transboundary diseases affecting the global swine industry. Its causative agent, African Swine Fever Virus (ASFV), is a large, complex, enveloped double-stranded DNA virus and the sole member of the *Asfivirus* genus within the *Asfarviridae* family [1,2,3]. Historically confined to sub-Saharan Africa—where the disease was first described in Kenya in 1921 and maintained within a sylvatic cycle involving warthogs and *Ornithodoros* soft ticks—the global distribution of ASF shifted dramatically in 2007 following the virus’s introduction into Georgia. This event marked the onset of an inexorable pan-continental spread [4]. Since 2018, the world has experienced an unprecedented epizootic characterized by rapid dissemination across Asia, particularly in China, Vietnam, and the Philippines, alongside persistent establishment in wild boar populations in Eastern and Central Europe [5]. This expansive spread has caused catastrophic economic losses, leading to the death or culling of hundreds of millions of pigs and posing serious threats to global pork production and food security [6]. The recent re-emergence of ASFV in the Americas—specifically in the Dominican Republic and Haiti in 2021 after nearly four decades of absence—further underscores the virus’s relentless transboundary potential and its ongoing threat to all swine-producing nations [7]. Consequently, ASF is designated as a notifiable disease by the World Organisation for Animal Health (WOAH), and its control remains an urgent global priority [8].

The control of ASF is remains exceptionally challenging, largely due to the lack of a globally available, safe, and effective vaccine [9]. In addition, the virus is remarkably resilient, capable of surviving for prolonged periods in the environment, on contaminated fomites, and in various pork products. Transmission is multifactorial, occurring through direct contact with infected domestic pigs, ingestion of contaminated feed, interaction with wild boar reservoirs, and persistence in survivor or carrier animals [5]. In the current absence of effective prophylactic or therapeutic tools, ASF control strategies depend almost entirely on “stamping-out” policies. These measures require rapid and early detection, strict on-farm biosecurity, movement restrictions, and the culling of affected and at-risk herds [10]. Consequently, timely and accurate diagnostics constitute the most critical pillar in the global response to ASF. Diagnostic approaches fall into two primary categories. The first is viral nucleic acid detection—most commonly performed using real-time quantitative PCR (qPCR), which remains the gold standard for identifying acutely infected animals during the viremic phase [11,12]. The second category includes WOAH-prescribed serological assays, such as ELISA and the immunoperoxidase test (IPT), which are used to identify animals that have mounted an antibody response [13].

While molecular detection by qPCR is essential for confirming acute outbreaks, serology remains indispensable for broader surveillance objectives, including proof-of-freedom certification, detection of chronic carriers or survivors, and monitoring low-virulence strains that may not produce overt clinical signs [14]. Despite their diagnostic value, both qPCR and conventional ELISA/IPT share a major operational limitation: they are laboratory-bound and time-consuming. Specifically, they require centralized facilities, expensive analytical equipment (such as thermal cyclers, plate readers, and washers), stable cold-chain logistics for reagent storage, and personnel with specialized technical training. Dependence on this infrastructure creates a diagnostic lag that often results in delays of several hours to days before results become available from the field. Such delays impede rapid containment efforts and are especially problematic in remote rural regions or in countries with limited veterinary diagnostic capacity [15]. Collectively, these limitations underscore the need for point-of-care (POC) diagnostic tools that are rapid, simple, cost-effective, and sufficiently robust for field or “pen-side” use without laboratory support. Lateral flow assays (LFAs), also referred to as immunochromatographic assays, meet these criteria by delivering results in under 20 min, requiring minimal operator training, and remaining stable at ambient temperatures [16].

In response to this need, numerous rapid LFAs for ASFV have been developed and commercialized [17,18]. Among the factors that influence their diagnostic performance, the most critical is the choice of recombinant antigen used to capture antibodies from the sample, as this directly determines test sensitivity and specificity. The ASFV proteome contains several highly immunodominant proteins that are widely targeted for serological assays. The most commonly used is the major capsid protein p72 (B646L), which is highly conserved, strongly immunogenic, and elicits a robust and persistent antibody response, making it a benchmark antigen in ASF serological assays [19]. In addition to P72, the p30 protein (CP204L) represents an important antigenic target; antibodies against p30 develop 2–4 days earlier than those against p72, positioning it as a valuable marker for early seroconversion [20,21]. A third widely recognized antigen is the p54 protein (E248R), an external envelope protein that is likewise highly immunogenic [22]. Although these antigens have been incorporated into duplex LFAs [23,24] and multi-antigen chimeric ELISAs [25], rigorous side-by-side comparisons of single-antigen LFAs remain limited. Consequently, it is still uncertain whether p54 contributes additional sensitivity or is susceptible to non-specific reactions in rapid assays, and whether a p30-based LFA performs as reliable as a p72-based LFA in detecting established infections. Clarifying the individual diagnostic contribution of each antigen is therefore critical for designing an optimal LFA. To address this gap, the present study developed and systematically evaluated three in-house single-antigen LFAs targeting p30, p54, and p72. By comparing their diagnostic sensitivity, specificity, and overall accuracy against a widely used commercial multi-antigen ELISA, this study aims to identify the most reliable antigen target for rapid, high-accuracy serological surveillance of ASFV.

## 2. Materials and Methods

### 2.1. Ethical Approval

The animal procedures were approved by the Animal Research Ethics Committee of the Faculty of Veterinary Medicine, Mahanakorn University of Technology, Thailand (Approval code: ACUC-MUT-2024/004).

### 2.2. Sample Collection and Characterization of the Study Population

A total of 143 swine serum samples were utilized as the testing panel for this study. To ensure the assessment of the LFAs was relevant to real-world surveillance scenarios in endemic regions, samples were collected from commercial swine farms located in high-risk areas of Thailand that had experienced sporadic ASF outbreaks. The study population was strictly limited to sows and replacement gilts (aged >6 months). This age group was selected to eliminate the potential interference of maternally derived (colostral) antibodies, which can confound serological results in piglets or weaners. Fattening pigs were excluded to focus on the breeding herd, which represents the long-term reservoir potential on farms.

**Figure 1:**
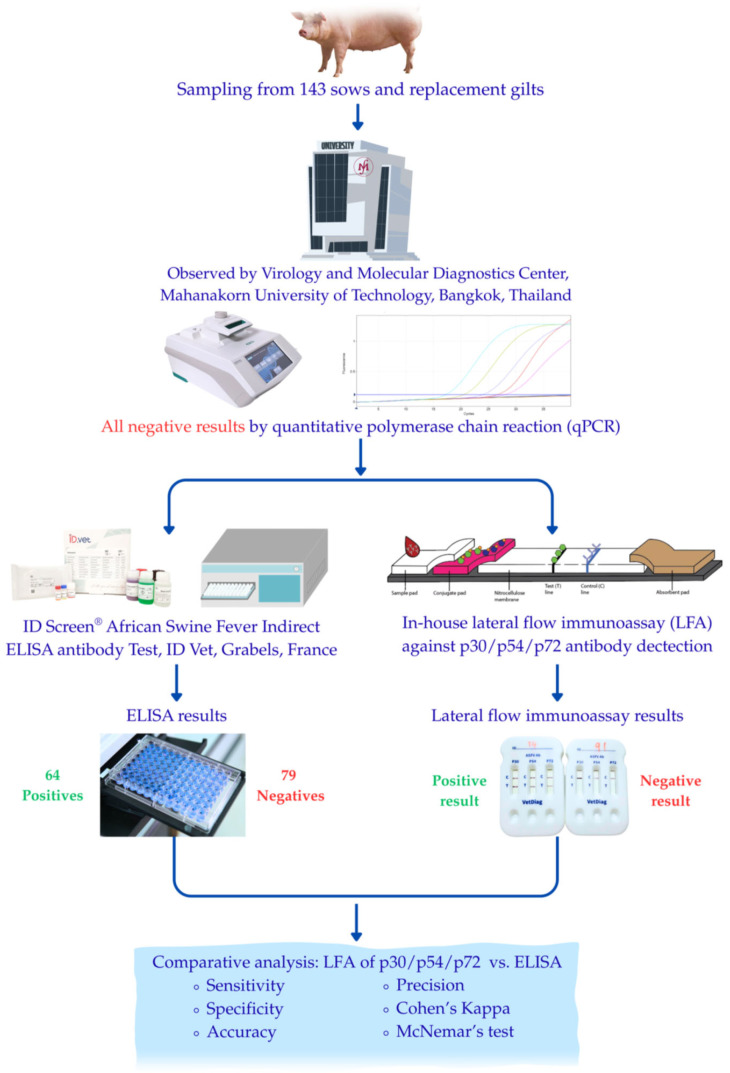
Schematic overview of the study design and diagnostic pipeline. One hundred forty-three swine serum samples, all confirmed negative for African swine fever virus (ASFV) by quantitative polymerase chain reaction (qPCR), were subjected to parallel comparative analysis. Samples were tested using the ID Screen^®^ African Swine Fever Indirect Antibody Test (ID Screen^®^, ID Vet, Grabels, France) and in-house lateral flow assays (LFAs) specific for p30, p54, and p72 antibody detection. The diagnostic performance of each LFA was statistically compared against the ELISA results to evaluate sensitivity, specificity, accuracy, and concordance.

At the time of sampling, all 143 animals were clinically healthy and showed no signs suggestive of ASF, such as fever, hemorrhage, or anorexia. To determine infection status, all samples were tested for ASFV DNA using a qPCR assay, and all were confirmed to be PCR-negative (Ct > 40). Following qPCR screening, the samples were further analyzed using a commercially available ELISA kit to determine their antibody status. Consequently, the seropositive animals identified in this study (*n* = 64) likely represent a population with prior exposure to ASFV. Although definitive classification would require longitudinal follow-up, this serological profile is consistent with convalescent animals or those that have recovered from infection, rather than acutely infected carriers. This completes a set of 143 well-characterized samples that served as the reference panel. All samples were obtained from the Virology and Molecular Diagnostic Center, Faculty of Veterinary Medicine, Mahanakorn University of Technology, Thailand, and were stored at−30 °C until testing (Figure 1).

### 2.3. Assessment of Analytical Specificity and Cross-Reactivity

To evaluate the analytical specificity of the developed LFAs, the assays were tested against serum samples positive for antibodies to other economically important swine pathogens that could potentially cause cross-reactivity or confound clinical diagnosis. The panel included sera positive for antibodies to Classical swine fever virus (CSFV), Porcine reproductive and respiratory syndrome virus (PRRSV), Foot-and-mouth disease virus (FMDV) serotype A, Porcine circovirus type 2 (PCV-2), and Pseudorabies virus (PRV-gB). These samples were obtained from the archive of the Virology and Molecular Diagnostic Center, Faculty of Veterinary Medicine, Mahanakorn University of Technology.

### 2.4. Quantitative Polymerase Chain Reaction

This protocol adhered to the methodological framework described in our previous study [26]. Briefly, viral DNA was extracted from all samples using the TAN Bead^®^ Nucleic Acid Extraction Kit (Taiwan Advanced Nanotech, Taoyuan, Taiwan) in conjunction with the Automated Nucleic Acid Extractor (Smart LabAssist SLA-E13200, Taoyuan, Taiwan). Following DNA extraction, quantitative detection of ASFV DNA was performed using the Virotype^®^ ASFV 2.0 PCR Kit (Indical Bioscience, Leipzig, Germany) on a C1000 Touch Thermal Cycler (Bio-Rad, Hercules, CA, USA). The assay incorporated two internal controls—an endogenous β-actin control and an exogenous control introduced during DNA purification—to ensure both extraction efficiency and amplification reliability. The qPCR cycling conditions were as follows: an initial denaturation at 95 °C for 2 min, followed by 40 cycles of 95 °C for 5 s and 60 °C for 30 s. Sample results were interpreted according to the manufacturer’s recommended cycle threshold (Ct) cutoffs: positive when Ct < 35, suspect when 35–40, and negative when >40.

### 2.5. Enzyme-Linked Immunosorbent Assay

All 143 swine serum samples were analyzed using a commercial indirect ELISA kit (ID Screen^®^, ID Vet, Grabels, France), which served as the reference method for this study. The kit’s microplate wells are coated with recombinant ASFV proteins p32, p62, and p72. The assay was performed following the manufacturer’s instructions. Briefly, all reagents and samples were equilibrated to room temperature (21 °C ± 5 °C). A total of 190 µL of Dilution Buffer was added to each well, followed by 10 µL of the Negative Control, Positive Control, or each test serum sample. The plate was then covered and incubated for 45 ± 4 min at 21 °C (±5 °C).

After incubation, the wells were emptied and washed three times with at least 300 µL of 1× Wash Solution. Subsequently, 100 µL of 1× anti-multi-species HRP conjugate—prepared by diluting the 10× concentrate 1:10 in Dilution Buffer—was added to each well, and the plate was incubated for an additional 30 ± 3 min at 21 °C (±5 °C). A second washing step identical to the first was then performed.

To initiate the colorimetric reaction, 100 µL of Substrate Solution was added to each well, and the plate was incubated in the dark for 15 ± 2 min at 21 °C (±5 °C). The reaction was stopped by adding 100 µL of Stop Solution (0.5 M acid), and the optical density (OD) of each well was immediately measured at 450 nm using a microplate reader (EUROIMMUN Analyzer I-2P, EUROIMMUN US, Inc., Mountain Lakes, NJ, USA).

The assay run was considered valid when the mean Positive Control OD (ODpc) exceeded 0.350 and the ODpc/ODnc ratio was greater than 3. For each sample, a sample-to-positive (S/P) percentage was calculated using the following formula:

*S/P%* = *[(ODsample* − *ODnc)*/*(ODpc*−*ODnc)]* × *100*

Samples with S/P% ≥ 40% were classified as positive, S/P% ≤ 30% as negative, and values between 30% and 40% were considered doubtful.

### 2.6. Lateral Flow Assay

The in-house LFA for ASFV antibody detection was developed and optimized based on a double recognition (indirect sandwich) assay principle. The finalized prototypes were manufactured by Pacific Biotech Co., Ltd. (Petchaboon, Thailand) under a controlled manufacturing process to ensure reproducibility. Briefly, the strip components were prepared as follows. For the Test line (T line), recombinant p30, p54, or p72 antigen was diluted in Tris-HCl (pH 8.5) containing sucrose and dispensed onto a nitrocellulose membrane. For the Control line (C line), a monoclonal antibody (MAb) specific to a non-relevant control protein was diluted in 20 mM Tris-HCl (pH 7.5) with sucrose and dispensed parallel to the T line on the same membrane. Detector reagents were prepared by coupling proteins to colloidal gold nanoparticles. Two separate conjugations were performed to allow differential detection: recombinant p30, p54, or p72 antigen was conjugated to one batch of nanoparticles, while the control protein recognized by the C-line MAb was conjugated to a second batch.

**Figure 2:**
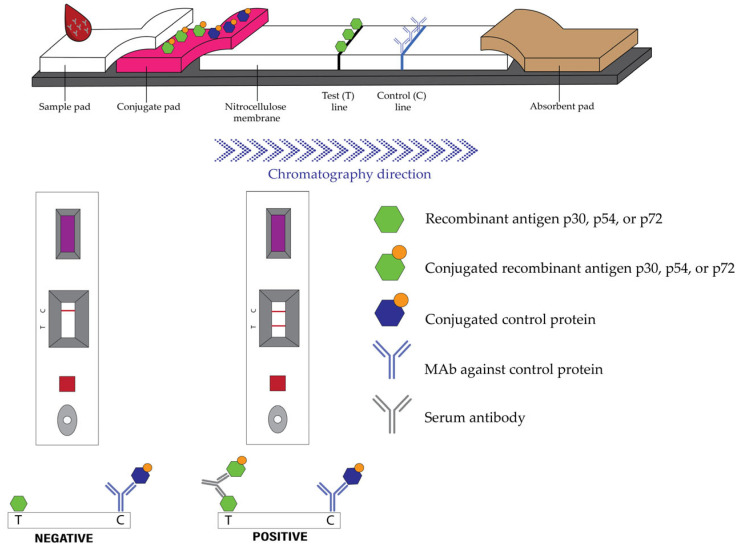
Principle of the in-house lateral flow assay (LFA) for ASFV antibody detection. In a positive sample, ASFV-specific serum antibodies (gray Y) bind simultaneously to the colloidal gold-conjugated recombinant antigen (e.g., p30, p54, or p72; green hexagon with orange circle) released from the conjugate pad and to the immobilized recombinant antigen (green hexagon) at the Test (T) line. This antigen–antibody sandwich complex generates a visible signal at the T line. In a negative sample, the colloidal gold-conjugated antigen migrates across the strip without binding and therefore does not produce a T-line signal. The Control (C) line, which must be present for the assays to be considered valid, contains an immobilized monoclonal antibody (blue Y) that captures a separate conjugated recombinant protein (blue pentagon with orange circle). The appearance of this C-line signal confirms adequate sample migration and the integrity of the assay reagent.

**Figure 3:**
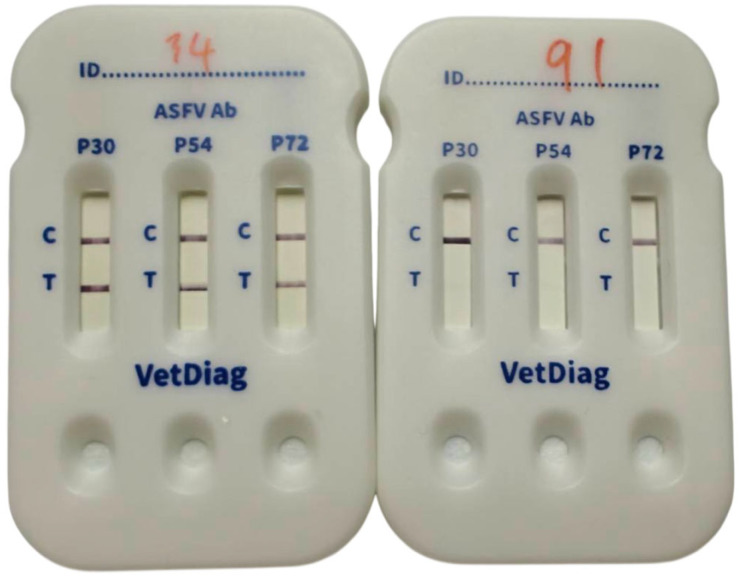
Examples of test results obtained using the in-house triple-strip LFA cassette. The cassette on the left shows a valid positive result for all three ASFV antibodies (P30, P54, and P72), with visible lines present at both the Control (C) and Test (T) positions on each corresponding strip. In contrast, the cassette on the right shows a valid negative result, in which only the C-line signals are visible, confirming proper assay performance but indicating the absence of detectable ASFV-specific antibodies.

The LFA strips were assembled by affixing the nitrocellulose membrane, conjugate pad, absorbent pad, and sample pad onto an adhesive backing card with appropriate overlaps (Figure 2). For the test procedure, 10 µL of serum or 20 µL of whole blood was applied to the sample window. After absorption, five drops (approximately 150 µL) of running buffer were added to the sample window, and the results were read after 10 min. A valid test was indicated by a visible signal at the C line, confirming proper sample migration and correct reagent functionality, as the gold-conjugated control protein was captured by the immobilized Mab (Figure 3).

### 2.7. Statistic Analysis

The diagnostic performance of the three in-house LFAs targeting P30, P54, and P72 was evaluated against a commercial indirect ELISA, which was considered the reference standard. The dichotomous outcome (positive/negative) from each LFA and the ELISA for all 143 serum samples were organized into 2 × 2 contingency tables, from which the numbers of true positives (TP), true negatives (TN), false positives (FP), and false negatives (FN) were obtained for each LFA prototype (Table 1).

Key diagnostic parameters were then calculated following established methods [27,28,29], including diagnostic sensitivity [TP / (TP + FN) × 100], diagnostic specificity [TN / (TN + FP) × 100], and overall diagnostic accuracy [(TP + TN) / (TP + TN + FP + FN) × 100]. In addition, the positive predictive value (PPV), also referred to as precision, was calculated using the formula [TP / (TP + FP) × 100] to estimate the likelihood that a positive LFA result represented a true positive. The 95% confidence intervals (CIs) for all diagnostic estimates were also computed.

To further assess the agreement between the LFAs and the reference method, two statistical tests were performed. Inter-rater concordance was evaluated using Cohen’s Kappa (κ), which measures the degree of agreement beyond chance. κ values were interpreted using standard criteria, where κ = 1.0 indicates perfect agreement and values > 0.81 indicate almost perfect agreement. McNemar’s test for paired nominal data was used to analyze discordant classifications (FP and FN) and to determine whether a significant difference or systematic bias existed between the LFAs and the ELISA. A *p*-value < 0.05 was considered statistically significant.

## 3. Results

### 3.1. Overall Sample Analysis

A total of 143 swine serum samples were included in the comparative analysis. All samples were confirmed negative for ASFV genetic material by qPCR prior to serological testing. The reference indirect ELISA was then used to determine the antibody status of these samples. Among them, 64 samples tested positive and 79 tested negatives for ASFV antibodies. Based on the ELISA results, the samples were subsequently tested using three prototype LFAs targeting the ASFV P30, P54, and P72 proteins, respectively.

### 3.2. Assay Performance and Contingency Analysis

The comparative performance of the three LFAs against the reference ELISA is summarized in the 2 × 2 contingency tables (Table 1). Detailed individual sample results are provided in the Supplementary Materials (Tables S1–S3).

The P72-based LFA demonstrated perfect concordance with the ELISA, correctly identifying all 64 positive samples (true positives, TP) and all 79 negative samples (true negatives, TN), with no false positives (FP = 0) or false negatives (FN = 0).

The P30-based LFA correctly identified all 64 positive samples (TP) and 78 of the 79 negative samples (TN). A single serum sample (Sample 13) produced a false-positive result (FP = 1), while no false negatives were observed (FN = 0).

The P54-based LFA correctly identified all 64 positive samples (TP); however, nine false positives were recorded (FP = 9), and 70 of the 79 negative samples were correctly classified (TN). No false negatives were observed (FN = 0).

### 3.3. Diagnostic Parameters and Statistical Agreement

The diagnostic sensitivity, specificity, accuracy, and precision (positive predictive value, PPV) for each assay were calculated from the contingency tables, and statistical agreement was further evaluated using Cohen’s Kappa (κ) and McNemar’s test (Table 2).

The LFA targeting P72 exhibited perfect performance, with 100% diagnostic sensitivity (95% CI: 94.4–100), 100% specificity (95% CI: 95.4–100), and 100% accuracy (95% CI: 97.5–100). Agreement with ELISA was perfect, with a Kappa value of 1.0.

The LFA targeting P30 also showed high performance, with 100% diagnostic sensitivity (95% CI: 94.4–100), 98.7% specificity (95% CI: 93.2–99.9), and 99.3% accuracy (95% CI: 96.4–99.9). Agreement with ELISA was classified as ‘Almost Perfect’ (κ = 0.9859), and McNemar’s test indicated no statistically significant difference between the P30 LFA and ELISA results (*p* > 0.999).

In contrast, the LFA targeting P54 achieved 100% diagnostic sensitivity (95% CI: 94.4–100) but had a lower specificity of 88.6% (95% CI: 79.4–94.7), resulting in an overall accuracy of 93.7% (95% CI: 88.5–96.9). Although the Kappa value indicated ‘Almost Perfect’ agreement (κ = 0.8745), McNemar’s test revealed a statistically significant difference (*p* = 0.0039) compared with ELISA, primarily due to the high rate of false positive results.

### 3.4. Cross-Reactivity Analysis

The analytical specificity of the P30, P54, and P72 LFAs was assessed using serum samples positive for antibodies to other common swine pathogens, including CSFV, PRRSV, FMDV serotype A, PCV-2, and PRV-gB. No cross-reactivity was detected, as all three LFA prototypes yielded negative results for every heterologous antibody tested. These findings demonstrate that the developed LFAs exhibit high analytical specificity for ASFV antibodies and do not cross-react with antibodies to these major swine viral pathogens.

## 4. Discussion

The global persistence of ASF underscores the need for rapid, accurate, and field-deployable serological diagnostics, which are crucial for effective disease management. Such tools are indispensable for surveillance, monitoring control measures, and certifying disease-free status, and complement early molecular detection methods [1]. Beyond antigen-specific performance, LFAs, as demonstrated by our P72 and P30 prototypes, offer key operational advantages over conventional serological methods such as ELISA or IPT. While laboratory-based assays require specialized equipment (e.g., plate readers, washers), cold-chain logistics, and trained personnel, LFAs are designed for field deployment. They are rapid, require minimal training for “pen-side” application, and remain stable at ambient temperatures. Another practical benefit of LFAs is their single-sample format. Unlike ELISA, which is most economical when run in batches (e.g., 96-well plates), an LFA can test a single, high-priority animal immediately and cost-effectively. This capability is crucial for rapid screening in smallholder settings, outbreak investigations, or movement control, providing actionable results in under 20 min rather than hours or days. In this study, we evaluated the diagnostic performance of three single-antigen LFAs targeting ASFV P30, P54, and P72, comparing them against a commercial multi-antigen indirect ELISA that targets recombinant P32, P62, and P72 proteins.

The LFA targeting P72 demonstrated perfect sensitivity and specificity (100%) and achieved complete agreement (κ = 1.0) with the reference ELISA, highlighting its exceptional diagnostic performance. This high performance was, to some extent, anticipated given the biological characteristics of the P72 protein. P72, encoded by the B646L gene, is the major, highly conserved capsid protein and the primary immunodominant antigen in ASFV infection [25,30,31]. Consequently, it serves as the benchmark target for many recommended serological tests, including the OIE-recognized immunoperoxidase test (IPT) [13]. Importantly, our single-antigen P72 LFA performed identically to the multi-antigen (P32/P62/P72) commercial ELISA. This indicates that, for the 143 samples in this panel, the anti-P72 antibody response was sufficiently robust and persistent to correctly identify all positive cases. Notably, the inclusion of P32 and P62 antigens in the ELISA, also known immunogenic proteins [32,33], provided no additional sensitivity in this dataset.

Furthermore, our single-antigen P30 LFA demonstrated excellent performance, with 100% sensitivity and 98.7% specificity. The single false positive (Sample 13) represents an acceptable discrepancy for a rapid screening test. These results are supported by the ‘Almost Perfect’ Kappa agreement (κ = 0.9859) and a non-significant McNemar’s test (*p* > 0.999). Biologically, the P30 protein (CP204L gene) is a well-established early diagnostic marker, with antibodies often appearing 2–4 days earlier than those against P72 [18,23,34]. Notably, our P30 LFA detected all 64 positive samples that were also identified by the P32/P62/P72-based ELISA. This concordance suggests that the samples were not in the very early seroconversion window (e.g., 7–10 days post-infection), a period when P30 might be the only detectable antibody [35]. Therefore, the dataset likely represents animals in the mid-to-late or chronic stages of infection, during which robust antibody populations against both P30 and P72 co-exist [36].

Conversely, the P54-based LFA was determined to be unsuitable for diagnostic use. Despite achieving 100% sensitivity, its specificity was only 88.6%. This resulted in nine false positives, which are unacceptable for any reliable surveillance program. This significant discrepancy (McNemar’s *p* = 0.0039) underscores a critical limitation of the assay. Although the P54 protein (E248R gene) is immunogenic [37], it may be prone to non-specific binding or cross-reactivity when used as a standalone target in a rapid lateral flow format, a limitation also noted in other studies [34]. While P54 has been used effectively in multi-antigen or chimeric ELISA formats [25,38], these results demonstrate its unreliability as a single target in an LFA.

A critical observation in this study was the high seroprevalence (44.7%, 64/143) identified within a PCR-negative, clinically healthy population. The presence of PCR-negative but seropositive animals in endemic regions represents a complex epidemiological scenario. Although such animals are often referred to in the field as “survivors,” this classification should be interpreted with caution. These animals show evidence of prior exposure and have mounted an immune response, yet they lacked detectable viral DNA in serum at the time of sampling. This profile clearly differs from that of acutely infected animals, which are typically qPCR-positive. Although seropositive animals may not contribute substantially to viral spread during their non-viremic phase, they serve as important sentinels indicating prior herd exposure to the virus.

It is crucial to consider the infection timeline when interpreting these results. The LFAs validated in this study are designed to detect antibodies, which typically become detectable 7–10 days post-infection. Accordingly, these assays are not suitable for identifying early, acute infections during the viremic phase prior to seroconversion. Consequently, these LFAs should not replace qPCR but should instead be used as complementary diagnostic tools. In a comprehensive surveillance program, qPCR is essential for detecting early outbreaks, whereas LFAs are valuable for retrospective screening and assessing herd immune status.

In designing the study, we specifically excluded piglets and weaners to eliminate the confounding effects of maternal antibodies, which can persist for several weeks. By restricting the sample set to sows and replacement gilts, we ensured that detected antibodies reflected active immune responses to natural field exposure.

The primary limitation of this study is the exclusion of PCR-positive (viremic) animals, fattening pigs, and time-course sera from experimentally infected individuals. Because the study’s objective was to validate the LFA’s sensitivity in detecting non-viremic survivors—the most diagnostically challenging group—the data could not conclusively confirm the expected early—phase detection advantage of P30 over P72. Controlled infection studies are therefore needed to precisely define the temporal diagnostic sensitivity of these assays.

Importantly, a distinction must be drawn between the analytical validation performed in this study and full field deployment. While the LFAs are designed for pen-side use, testing in the present study was conducted under controlled laboratory conditions using stored serum samples. Although the assay format is rapid and requires no equipment, performance under field conditions may be influenced by factors such as extreme environmental temperatures, dust exposure, and interpretation by non-technical personnel. Therefore, additional field testing across diverse environmental conditions is recommended to confirm the practical robustness of these LFAs.

Although the laboratory validation performed here was rigorous, additional field testing across diverse environmental conditions is recommended to confirm the practical robustness of these LFAs. As PCR remains the gold standard for detecting acute or viremic infections, the main utility of LFAs lies in identifying chronic or convalescent cases in which PCR results are negative. Future research should include PCR-positive animals and fattening pigs to correlate LFA reactivity with viral load dynamics and clinical progression.

Finally, the analytical specificity of the LFAs was clearly demonstrated by the absence of cross-reactivity with major swine antibodies, including CSFV, PRRSV, FMDV serotype A, PCV-2, and PRV-gB. This high specificity is particularly critical for differentiating ASF from CSFV, which presents with clinically indistinguishable hemorrhagic signs, ensuring that positive LFA results reliably indicate ASFV exposure.

Based on these findings, these results strongly support the development of a duplex LFA. Such a test, combining P30 and P72 on a single strip as previously described by others [24,39], would leverage the strengths of both markers. Specifically, it would utilize the P30 antigen to detect antibodies in the critical early serological window and the P72 antigen to ensure robust detection of persistent antibodies in mid-to-late stages. This tool would theoretically provide diagnostic coverage superior to our single-antigen prototypes and could match or exceed the utility of the multi-antigen (P32/P62/P72) ELISA in a rapid, field-deployable format.

Finally, the demonstrated diagnostic performance of these assays has important implications for global ASF control strategies. According to the Global Framework for the Progressive Control of Transboundary Animal Diseases (GF-TADs), rapid and decentralized diagnostic tools are essential for effective disease management, particularly in resource-limited settings [26]. By offering a reliable and low-cost alternative to laboratory-bound assays, the P72 and P30 LFAs described here can strengthen surveillance capacity, facilitate faster field-level response by local veterinarians, and help maintain disease-free zones in endemic regions.

## 5. Conclusions

This study demonstrated high diagnostic performance of single-antigen LFAs targeting ASFV P72 and P30. The P72 LFA exhibited perfect statistical agreement, while the P30 LFA showed almost perfect agreement with a commercial multi-antigen ELISA reference. The P72 LFA proved robust for detecting established antibody responses, whereas the P30 LFA served as a reliable serological marker. In contrast, the P54 LFA was deemed unsuitable for diagnostic use due to unacceptably low specificity. Taken together, these findings indicate that the P72 and P30 LFAs are highly accurate candidates for use in serosurveillance. However, they should be interpreted as assays for past exposure and applied as complementary tools alongside molecular methods for acute infection detection.

## Figures and Tables

**Table 1:** Performance of antibody detection against P30, P54, and P72 of African swine fever virus using lateral flow assays (LFAs) compared to an enzyme-linked immunosorbent assay (ELISA).

		ELISA	
		Positive	Negative
P30	Positive	64	1
	Negative	0	78
P54	Positive	64	9
	Negative	0	70
P72	Positive	64	0
	Negative	0	79

**Table 2:** Analytical sensitivity, specificity, accuracy, precision, corresponding 95% confidence intervals, Cohen’s Kappa, and McNemar’s test for lateral flow assays (LFAs) targeting various structural proteins of African swine fever virus (ASFV) compared to a commercial enzyme-linked immunosorbent assay (ELISA).

Test	Parameter	Value (%) (95% CI)	Cohen’s Kappa	McNemar’s Test
P30	Sensitivity	100 (94.4–100)	κ = 0.9859	*p* > 0.999
	Specificity	98.7 (93.2–99.9)		
	Accuracy	99.3 (96.4–99.9)		
	Precision	98.5 (91.8–99.9)		
P54	Sensitivity	100 (94.4–100)	κ = 0.8745	*p* = 0.0039
	Specificity	88.6 (79.4–94.7)		
	Accuracy	93.7 (88.5–96.9)		
	Precision	87.7 (77.9–94.0)		
P72	Sensitivity	100 (94.4–100)	κ = 1.0	
	Specificity	100 (95.4–100)		
	Accuracy	100 (97.5–100)		
	Precision	100 (94.4–100)		

## Data Availability

The original contributions presented in this study are included in the article/supplementary material. Further inquiries can be directed to the corresponding author.

## Supplementary Materials

The following supporting information can be downloaded at: https://www.mdpi.com/article/10.3390/vetsci13010004/s1, Table S1. Individual results of antibody detection against African swine fever virus P30 obtained using a lateral flow assay (LFA) and compared with an enzyme-linked immunosorbent assay (ELISA). Table S2. Individual results of antibody detection against African swine fever virus P54 obtained using a lateral flow assay (LFA) and compared with an enzyme-linked immunosorbent assay (ELISA). Table S3. Individual results of antibody detection against African swine fever virus P72 obtained using a lateral flow assay (LFA) and compared with an enzyme-linked immunosorbent assay (ELISA).
