# Supplementary material for: High-Accuracy Serodiagnosis of African Swine Fever Using P72 and P30-Based Lateral Flow Assays: A Validation Study with Field Samples in Thailand
Source: Vet Sci. 2025 Dec 19;13(1):4. doi: 10.3390/vetsci13010004 (PMC12846366; doi:10.3390/vetsci13010004)
Supplement: Supplementary file 1 [file vetsci-13-00004-s001.zip › vetsci-4019246-supplementary.pdf]

Table-S1. Individual results of antibody detection against African swine fever virus P30 obtained using a lateral flow assay (LFA) and compared with an enzyme-linked immunosorbent assay (ELISA).

No.	LFA	ELISA	No.	LFA	ELISA	No.	LFA	ELISA	No.	LFA	ELISA
1	Negative	Negative	37	Positive	Positive	73	Negative	Negative	109	Negative	Negative
2	Positive	Positive	38	Positive	Positive	74	Negative	Negative	110	Negative	Negative
3	Negative	Negative	39	Positive	Positive	75	Negative	Negative	111	Negative	Negative
4	Negative	Negative	40	Positive	Positive	76	Negative	Negative	112	Positive	Positive
5	Negative	Negative	41	Positive	Positive	77	Negative	Negative	113	Positive	Positive
6	Negative	Negative	42	Positive	Positive	78	Negative	Negative	114	Positive	Positive
7	Negative	Negative	43	Positive	Positive	79	Negative	Negative	115	Positive	Positive
8	Negative	Negative	44	Positive	Positive	80	Negative	Negative	116	Positive	Positive
9	Negative	Negative	45	Negative	Negative	81	Negative	Negative	117	Positive	Positive
10	Negative	Negative	46	Negative	Negative	82	Negative	Negative	118	Positive	Positive
11	Negative	Negative	47	Negative	Negative	83	Negative	Negative	119	Positive	Positive
12	Negative	Negative	48	Negative	Negative	84	Negative	Negative	120	Positive	Positive
13	Positive	Negative	49	Negative	Negative	85	Negative	Negative	121	Positive	Positive
14	Positive	Positive	50	Negative	Negative	86	Negative	Negative	122	Positive	Positive
15	Positive	Positive	51	Negative	Negative	87	Negative	Negative	123	Positive	Positive
16	Positive	Positive	52	Negative	Negative	88	Negative	Negative	124	Positive	Positive
17	Positive	Positive	53	Negative	Negative	89	Negative	Negative	125	Positive	Positive
18	Positive	Positive	54	Negative	Negative	90	Negative	Negative	126	Positive	Positive
19	Positive	Positive	55	Negative	Negative	91	Negative	Negative	127	Positive	Positive
20	Positive	Positive	56	Negative	Negative	92	Negative	Negative	128	Positive	Positive
21	Positive	Positive	57	Negative	Negative	84	Negative	Negative	129	Positive	Positive
22	Positive	Positive	58	Negative	Negative	94	Negative	Negative	130	Positive	Positive
23	Positive	Positive	59	Negative	Negative	95	Negative	Negative	131	Positive	Positive
24	Positive	Positive	60	Negative	Negative	96	Negative	Negative	132	Positive	Positive
25	Positive	Positive	61	Negative	Negative	97	Negative	Negative	133	Positive	Positive
26	Positive	Positive	62	Negative	Negative	98	Negative	Negative	134	Positive	Positive
27	Positive	Positive	63	Negative	Negative	99	Negative	Negative	135	Positive	Positive
28	Positive	Positive	64	Negative	Negative	100	Negative	Negative	136	Positive	Positive
29	Positive	Positive	65	Negative	Negative	101	Negative	Negative	137	Positive	Positive
30	Positive	Positive	66	Negative	Negative	102	Negative	Negative	138	Positive	Positive
31	Positive	Positive	67	Negative	Negative	103	Negative	Negative	139	Positive	Positive
32	Positive	Positive	68	Negative	Negative	104	Negative	Negative	140	Positive	Positive
33	Positive	Positive	69	Negative	Negative	105	Negative	Negative	141	Positive	Positive
34	Positive	Positive	70	Negative	Negative	106	Negative	Negative	142	Positive	Positive
35	Positive	Positive	71	Negative	Negative	107	Negative	Negative	143	Positive	Positive
36	Positive	Positive	72	Negative	Negative	108	Negative	Negative			

Table-S2. Individual results of antibody detection against African swine fever virus P54 obtained using a lateral flow assay (LFA) and compared with an enzyme-linked immunosorbent assay (ELISA).

No.	LFA	ELISA	No.	LFA	ELISA	No.	LFA	ELISA	No.	LFA	ELISA
1	Positive	Negative	37	Positive	Positive	73	Negative	Negative	109	Negative	Negative
2	Positive	Positive	38	Positive	Positive	74	Negative	Negative	110	Negative	Negative
3	Positive	Negative	39	Positive	Positive	75	Negative	Negative	111	Positive	Negative
4	Positive	Negative	40	Positive	Positive	76	Negative	Negative	112	Positive	Positive
5	Negative	Negative	41	Positive	Positive	77	Negative	Negative	113	Positive	Positive
6	Positive	Negative	42	Positive	Positive	78	Negative	Negative	114	Positive	Positive
7	Negative	Negative	43	Positive	Positive	79	Negative	Negative	115	Positive	Positive
8	Negative	Negative	44	Positive	Positive	80	Negative	Negative	116	Positive	Positive
9	Positive	Negative	45	Negative	Negative	81	Negative	Negative	117	Positive	Positive
10	Positive	Negative	46	Negative	Negative	82	Negative	Negative	118	Positive	Positive
11	Negative	Negative	47	Negative	Negative	83	Negative	Negative	119	Positive	Positive
12	Positive	Negative	48	Negative	Negative	84	Negative	Negative	120	Positive	Positive
13	Positive	Negative	49	Negative	Negative	85	Negative	Negative	121	Positive	Positive
14	Positive	Positive	50	Negative	Negative	86	Negative	Negative	122	Positive	Positive
15	Positive	Positive	51	Negative	Negative	87	Negative	Negative	123	Positive	Positive
16	Positive	Positive	52	Negative	Negative	88	Negative	Negative	124	Positive	Positive
17	Positive	Positive	53	Negative	Negative	89	Negative	Negative	125	Positive	Positive
18	Positive	Positive	54	Negative	Negative	90	Negative	Negative	126	Positive	Positive
19	Positive	Positive	55	Negative	Negative	91	Negative	Negative	127	Positive	Positive
20	Positive	Positive	56	Negative	Negative	92	Negative	Negative	128	Positive	Positive
21	Positive	Positive	57	Negative	Negative	84	Negative	Negative	129	Positive	Positive
22	Positive	Positive	58	Negative	Negative	94	Negative	Negative	130	Positive	Positive
23	Positive	Positive	59	Negative	Negative	95	Negative	Negative	131	Positive	Positive
24	Positive	Positive	60	Negative	Negative	96	Negative	Negative	132	Positive	Positive
25	Positive	Positive	61	Negative	Negative	97	Negative	Negative	133	Positive	Positive
26	Positive	Positive	62	Negative	Negative	98	Negative	Negative	134	Positive	Positive
27	Positive	Positive	63	Negative	Negative	99	Negative	Negative	135	Positive	Positive
28	Positive	Positive	64	Negative	Negative	100	Negative	Negative	136	Positive	Positive
29	Positive	Positive	65	Negative	Negative	101	Negative	Negative	137	Positive	Positive
30	Positive	Positive	66	Negative	Negative	102	Negative	Negative	138	Positive	Positive
31	Positive	Positive	67	Negative	Negative	103	Negative	Negative	139	Positive	Positive
32	Positive	Positive	68	Negative	Negative	104	Negative	Negative	140	Positive	Positive
33	Positive	Positive	69	Negative	Negative	105	Negative	Negative	141	Positive	Positive
34	Positive	Positive	70	Negative	Negative	106	Negative	Negative	142	Positive	Positive
35	Positive	Positive	71	Negative	Negative	107	Negative	Negative	143	Positive	Positive
36	Positive	Positive	72	Negative	Negative	108	Negative	Negative			

Table-S3. Individual results of antibody detection against African swine fever virus P72 obtained using a lateral flow assay (LFA) and compared with an enzyme-linked immunosorbent assay (ELISA).

No.	LFA	ELISA	No.	LFA	ELISA	No.	LFA	ELISA	No.	LFA	ELISA
1	Negative	Negative	37	Positive	Positive	73	Negative	Negative	109	Negative	Negative
2	Positive	Positive	38	Positive	Positive	74	Negative	Negative	110	Negative	Negative
3	Negative	Negative	39	Positive	Positive	75	Negative	Negative	111	Negative	Negative
4	Negative	Negative	40	Positive	Positive	76	Negative	Negative	112	Positive	Positive
5	Negative	Negative	41	Positive	Positive	77	Negative	Negative	113	Positive	Positive
6	Negative	Negative	42	Positive	Positive	78	Negative	Negative	114	Positive	Positive
7	Negative	Negative	43	Positive	Positive	79	Negative	Negative	115	Positive	Positive
8	Negative	Negative	44	Positive	Positive	80	Negative	Negative	116	Positive	Positive
9	Negative	Negative	45	Negative	Negative	81	Negative	Negative	117	Positive	Positive
10	Negative	Negative	46	Negative	Negative	82	Negative	Negative	118	Positive	Positive
11	Negative	Negative	47	Negative	Negative	83	Negative	Negative	119	Positive	Positive
12	Negative	Negative	48	Negative	Negative	84	Negative	Negative	120	Positive	Positive
13	Negative	Negative	49	Negative	Negative	85	Negative	Negative	121	Positive	Positive
14	Positive	Positive	50	Negative	Negative	86	Negative	Negative	122	Positive	Positive
15	Positive	Positive	51	Negative	Negative	87	Negative	Negative	123	Positive	Positive
16	Positive	Positive	52	Negative	Negative	88	Negative	Negative	124	Positive	Positive
17	Positive	Positive	53	Negative	Negative	89	Negative	Negative	125	Positive	Positive
18	Positive	Positive	54	Negative	Negative	90	Negative	Negative	126	Positive	Positive
19	Positive	Positive	55	Negative	Negative	91	Negative	Negative	127	Positive	Positive
20	Positive	Positive	56	Negative	Negative	92	Negative	Negative	128	Positive	Positive
21	Positive	Positive	57	Negative	Negative	84	Negative	Negative	129	Positive	Positive
22	Positive	Positive	58	Negative	Negative	94	Negative	Negative	130	Positive	Positive
23	Positive	Positive	59	Negative	Negative	95	Negative	Negative	131	Positive	Positive
24	Positive	Positive	60	Negative	Negative	96	Negative	Negative	132	Positive	Positive
25	Positive	Positive	61	Negative	Negative	97	Negative	Negative	133	Positive	Positive
26	Positive	Positive	62	Negative	Negative	98	Negative	Negative	134	Positive	Positive
27	Positive	Positive	63	Negative	Negative	99	Negative	Negative	135	Positive	Positive
28	Positive	Positive	64	Negative	Negative	100	Negative	Negative	136	Positive	Positive
29	Positive	Positive	65	Negative	Negative	101	Negative	Negative	137	Positive	Positive
30	Positive	Positive	66	Negative	Negative	102	Negative	Negative	138	Positive	Positive
31	Positive	Positive	67	Negative	Negative	103	Negative	Negative	139	Positive	Positive
32	Positive	Positive	68	Negative	Negative	104	Negative	Negative	140	Positive	Positive
33	Positive	Positive	69	Negative	Negative	105	Negative	Negative	141	Positive	Positive
34	Positive	Positive	70	Negative	Negative	106	Negative	Negative	142	Positive	Positive
35	Positive	Positive	71	Negative	Negative	107	Negative	Negative	143	Positive	Positive
36	Positive	Positive	72	Negative	Negative	108	Negative	Negative			
